# Cardiac magnetic resonance imaging-large language model Meta AI: a finetuned large language model for identifying findings and associated attributes in cardiac magnetic resonance imaging reports

**DOI:** 10.1016/j.jocmr.2025.101968

**Published:** 2025-11-13

**Authors:** Michelle Z. Fang, Makiya Nakashima, Kailash Singh, Eileen Galvani, Xiaotan Sun, Sharmeen Sorathia, Kevin Dorocak, Deborah Kwon, Christopher Nguyen, David Chen

**Affiliations:** a Cleveland Clinic Lerner College of Medicine at Case Western, Cleveland, Ohio, USA; b Heart Vascular and Thoracic Institute, Cleveland Clinic, Cleveland, Ohio, USA; c Cardiovascular Innovation Research Center, Cleveland Clinic, Cleveland, Ohio, USA; d Diagnostics Institute, Cleveland Clinic, Cleveland, Ohio, USA

**Keywords:** Transformers, Natural language processing, Ground-truth labeling, Named entity recognition, Diagnosis extraction, Findings extraction, Radiology report analysis

## Abstract

**Background:**

Cardiac magnetic resonance imaging (CMR) studies contain a wealth of information on a patient’s cardiovascular status. The ability to extract this data from free-text reports could serve to automate clinical decision support tools and generate data for retrospective clinical knowledge discovery, and clinical operational purposes. Few studies have examined the automatic extraction of data from free-text CMR reports, and the existing studies that do have key limitations, including small sample size and disease-specific data extraction. Existing studies also fail to extract features associated with the cardiovascular conditions that reflect nuances in natural language, such as uncertainty, severity, subtype, and anatomical locations of the condition. The goal of this study was to build a broad named entity recognition model to automatically extract a broad variety of common CMR findings and their associated attributes from CMR reports.

**Methods:**

We fine-tuned a Large Language Model Meta AI (LLaMA) model trained to identify 34 cardiovascular conditions and their associated attributes, including certainty, severity, location, and subtype of the condition. This model was trained on 1778 MRI reports and tested on 397 reports in an held-out test set and another 428 reports from another site in our hospital system with independent radiology practice and scanners.

**Results:**

Our model shows robust performance in predicting the mention of the 31 cardiovascular conditions (average F1 = 0.85). It also showed strong performance predicting attributes, including certainty (average F1 = 0.97) and severity (average F1 = 0.97). Model performance on the external validation set was generally slightly lower than the internal validation set, but performance was still strong (average F1 = 0.78 for mention, 0.97 for certainty, and 0.96 for severity).

**Conclusion:**

CMR-LLaMA has strong performance identifying a variety of concept mentions and moderate accuracies in extracting a selection of other associated attributes. NLP models can be used to automate the extraction of data from CMR reports to potentially assist with clinical and research workflow.

## Graphical abstract

**Figure:**
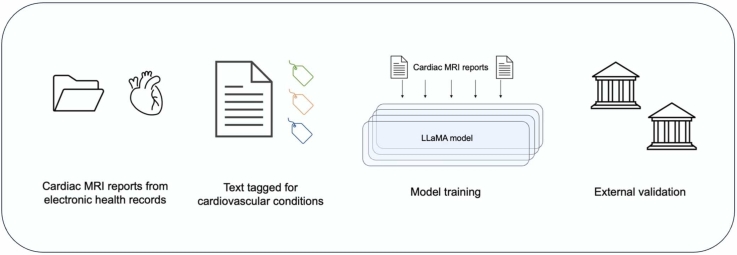

## 1 Introduction

Cardiovascular magnetic resonance imaging (CMR) is an important imaging modality to evaluate many cardiovascular conditions [1]. CMR is the gold standard for evaluating a wide variety of morphologic, functional, and tissue parameters, such as cardiac chamber volumes, function and mass, and extent of fibrosis [1]. Such information is used to inform management for a wide variety of diseases, including heart failure, ischemic disease, and valvular diseases [2], [3], [4]. Unfortunately, the vast amount of imaging features collected makes CMR reports a dense source of clinical information. Of which, only a subset of imaging findings are relevant for individual diseases, making effective reports balancing the concepts of completeness and conciseness difficult. The variation between individualized writing styles of these free-text reports further introduces room for errors in interpretation by the reader.

Automatic extraction of information in such free-text reports into a structured and standardized format can better inform downstream actionability of the study’s findings. Practically, structured and standardized medical documentation has been found to be easier to interpret by clinicians, achieving both improved completeness and conciseness [5], [6]. Many risk models dependent on these findings are currently manually integrated into care processes [7]. Automation of such risk scores has been shown to reduce cognitive burden and improve care [8]. Beyond its clinical utility, the ability to identify diagnoses from CMR reports can assist in clinical knowledge discovery through database construction.

However, few studies have examined the automatic extraction of data from free-text CMR reports. The existing models face key limitations, including limited sample size and the number of cardiovascular conditions able to be predicted. Dewaswala et al. examined the extraction of only hypertrophic cardiomyopathy from 391 CMR reports [9]. Zaman et al. only examined five cardiac diagnoses [10]. Algorithms used for extraction have included rule-based approaches [9] and conventional (non-neural network) machine learning algorithms [11], but not transformer-based pre-trained language models. Furthermore, these existing studies largely have not been able to identify attributes of the cardiovascular conditions that reflect nuances in natural language [9], [10]. This includes capturing uncertainty of the differential diagnosis, as well as severity, subtype, and anatomical location(s) of the condition.

In this study, we develop a large language model, which we dub CMR Large Language Model Meta AI (CMR-LLaMA), to automate the extraction of a broader number of common findings and disease diagnosis found in CMR reports. The model further automatically extracts the severity, subtype, pattern, and anatomic location of these findings to support both automating clinical risk models and retrospective clinical research. We hypothesize that the model achieves near-human-level performance for identifying clinical findings to enable scalable extraction of CMR data.

## 2 Methods

We trained CMR-LLaMA, a transformer-based model that extracts 31 cardiovascular conditions, along with attributes relating to those conditions including certainty, severity, location, and pattern. This model was trained on 2175 CMR reports, evaluated on 397 additional reports, then further externally validated on 428 additional reports from another hospital campus. This retrospective study was reviewed and approved by the Cleveland Clinic Institutional Review Board.

### 2.1 Annotation and note preprocessing

A total of 2200 CMR reports were extracted from patients of the Cleveland Clinic Main Campus’s electronic health records. Half the reports were randomly selected from a previously curated cohort of patients with nonischemic cardiomyopathies (NICM), including hypertrophic cardiomyopathy, cardiac amyloidosis, and non-differentiated NICM. The other half was randomly selected from all other studies.

Standard practice for radiology reports, including CMR reports, is to contain the following three main sections: 1) an “indications/clinical history” section which details the clinical context of the patient, 2) a “findings” section which comprehensively and objectively details findings in the study, and 3) an “impression” section which summarizes the more important findings relative to clinical decision making [12]. The impression section from each report was extracted for the analysis because of its key role in summarizing information about the imaging study and its nature of typically being free text. At our institution’s main campus, other sections are often filled in programmatically from the analytic software (e.g., table of biomarker measurements).

To generate the data for the model, three medical students and one resident were trained by a level-3 CMR reader to identify entities and their attributes. Annotators used MedTator to manually annotate the MRI text reports with a predetermined schema containing the 34 cardiovascular conditions and their associated features [13]. MedTator is an open source, serverless, text annotation tool designed to aid in corpus development [13]. The annotator can highlight the entity within the text and classify the selected text as one of pre-defined entities. An example of this process is shown in Supplemental Figure 1. The training set was equally split between the annotators, while the test set was annotated by every annotator. Inter-annotator agreement was measured using Fleiss' Kappa Score.

Differences between annotators were resolved using a voting system where the condition/attributes with the most votes decided the final annotation for that report. If there was a tie between attributes, the attribute would default to the more severe value. For example, if there was ambiguity between aortic dilation severity being ectatic or aneurysmal, the severity would default to aneurysmal. The left ventricular late gadolinium enhancement condition has an attribute for pattern (epicardial, mid-myocardial, subendocardial, transmural) and the aortic dilation condition has an attribute for location (root, ascending, descending). Due to the possibility of multiple of these attributes being simultaneously true (e.g., dilation of the aortic root and ascending aorta), all the patterns/locations with at least two annotators in agreement were included in the final test set.

Table 1 contains a summary of the annotated data characteristics that the model was trained on. Thirty-one findings relating to a variety of cardiovascular conditions were annotated. Each finding had a variety of attributes, including certainty (e.g., negated, possible, positive), severity (e.g., mild, moderate, severe), and location/subtype (e.g., ascending aorta, descending aorta, enhancement pattern). Findings with <30 instances of being used in the training dataset were excluded from the analysis due to insufficient sample size.

**Table 1:** Data characteristics for 2175 total CMR reports in the combined training and test datasets

	Condition	Certainty			Severity					
		Negated	Possible	Positive	Mild	Mild-moderate		Moderate	Moderate-Severe	Severe
Valve	Mitral valve regurgitation	20	0	594	328	69		123	30	20
	Aortic valve regurgitation	73	0	396	259	30		62	18	27
	Aortic valve stenosis	30	0	82	12	0		6	0	29
	Tricuspid valve regurgitation	8	0	194	111	21		37	0	14
	Pulmonary valve regurgitation	9	0	55	23	0		8	0	19
	Aortic valve bicuspid	16	0	136						
Heart chambers	RA dilation	0	0	209	117	NA		55	2	25
	LA dilation	205	2	266	139	NA		54	0	40
	RV dysfunction	143	8	336	173	NA		93	10	59
	RV dilation	0	0	316	136	NA		113	4	50
	LV dysfunction	333	2	631	247	NA		149	NA	144
	LV dilation	253	9	780	270	NA		223	NA	245
	LV hypertrophy	22	0	309	90	NA		73	NA	50
	LV late gadolinium enhancement	201	0	688	**Pattern**					
					**Epicardial**	**Mid-myocardial**		**Subendocardial**		**Transmural**
					6	31		19		28
	LV fibrosis	60	8	129						
	LV noncompaction	5	15	0						
Aorta					**Location**				**Severity**	
					**Root**	**Ascending**	**Arch**	**Descending**	**Ectatic**	**Aneurysmal**
	Aortic dilation	77	0	695	341	315	28	42	492	85
	Aortic atherosclerosis	2	8	38	0	0	6	32		
	Aortic arteritis	22	0	17						
Other	Hypertensive pulmonary disease	0	73	24						
	Hypertrophic cardiomyopathy	17	9	163						
	Infiltrative cardiomyopathy	94	37	27						
	Ischemic cardiomyopathy	43	21	137						
	Nonischemic cardiomyopathy	0	40	212						
	Myocardial infarction	19	20	106						
	Myocarditis/pericarditis	201	123	131						
	Papillary muscle thickening	45	0	79						
	Pericardial effusion	75	0	209						
	Pleural effusion	0	0	150						
	Sarcoidosis	43	84	22						
	Amyloidosis	28	36	44						

The number of instances of each of the 31 cardiovascular conditions and their associated features (certainty and severity) are shown. Cardiovascular conditions in which there were <30 mentions in the MRI reports were excluded from analysis (not shown). NA denotes attributes that were not applicable to that condition. For some certainty and/or severity levels, 0 indicates that there were no instances of this specific certainty/severity in the entire dataset. *LA* left atrium, *RA* right atrium, *LV* left ventricle, *RV* right ventricle, *CMR* cardiovascular magnetic resonance

**Table 2:** Inter-annotator agreement between four annotators each annotating the N = 397 test set

Condition	Mention	Certainty	Severity	Other
Mitral valve regurgitation	0.88	0.84	0.82	
Aortic valve regurgitation	0.84	0.76	0.82	
Aortic valve stenosis	0.83	0.75	0.48	
Tricuspid valve regurgitation	0.91	0.85	0.87	
Pulmonary valve regurgitation	0.80	0.70	0.80	
Aortic valve bicuspid	0.88	0.69		
RA dilation	0.73	0.73	0.73	
LA dilation	0.88	0.51	0.71	
RV dysfunction	0.80	0.50	0.68	
RV dilation	0.88	0.87	0.80	
LV dysfunction	0.88	0.57	0.76	
LV dilation	0.79	0.50	0.67	
LV hypertrophy	0.65	0.64	0.54	
LV late gadolinium enhancement	0.23	0.18		0.25
LV fibrosis	0.29	0.21		
LV noncompaction	0.38	0.36		
Aortic dilation	0.47	0.45		0.19
Aortic atherosclerosis	0.92	0.81	0.82	0.84
Aortic arteritis	0.45	0.45		
Hypertensive pulmonary disease	0.68	0.53		
Hypertrophic cardiomyopathy	0.83	0.80		
Infiltrative cardiomyopathy	0.54	0.30		
Ischemic cardiomyopathy	0.56	0.37		
Nonischemic cardiomyopathy	0.77	0.66		
Myocardial infarction	0.48	0.33		
Myocarditis/pericarditis	0.79	0.66		
Papillary muscle thickening	0.52	0.45		
Pericardial effusion	0.87	0.78		
Pleural effusion	0.61	0.59		
Sarcoidosis	0.81	0.75		
Amyloidosis	0.83	0.77		

Agreement was measured using the Fleiss' Kappa Score. The closer the score is to 1.0, the higher level of agreement. Scores are shown for the binary mention of 31 conditions and their attributes (certainty, severity, other). The other column refers to pattern for the condition LV late gadolinium enhancement (subendocardial, transmural, mid-myocardial, epicardial) and location for aortic dilation and aortic atherosclerosis (root, ascending, arch, and descending aorta). *LA* left atrium, *RA* right atrium, *LV* left ventricle, *RV* right ventricle

**Table 3:** Data characteristics for 428 CMR reports in the Cleveland Clinic Florida campus validation dataset

	Condition	Certainty			Severity					
		Negated	Possible	Positive	Mild	Mild-Moderate		Moderate	Moderate-Severe	Severe
Valve	Mitral valve regurgitation	11	6	71	41	7		11	1	1
	Aortic valve regurgitation	8	1	36	19	1		8	2	1
	Aortic valve stenosis	5	6	14	8	1		2	0	4
	Tricuspid valve regurgitation	1	0	30	12	7		3	5	0
	Pulmonary valve regurgitation	1	0	14	4	0		1	1	8
	Aortic valve bicuspid	5	0	8						
Heart chambers	RA dilation	0	0	23	11	NA		7	1	2
	LA dilation	85	0	42	17	NA		12	0	4
	RV dysfunction	175	2	88	46	NA		25	0	7
	RV dilation	2	0	49	19	NA		12	1	5
	LV dysfunction	70	1	84	46	NA		17	NA	7
	LV dilation	248	3	208	80	NA		47	NA	43
	LV hypertrophy	17	0	76	31	NA		10	NA	23
	LV late gadolinium enhancement	210	2	167	**Pattern**					
					**Epicardial**	**Mid-myocardial**		**Subendocardial**		**Transmural**
					3	44		11		33
	LV fibrosis	2	0	2						
	LV noncompaction	0	3	2						
Aorta					**Location**				**Severity**	
					**Root**	**Ascending**	**Arch**	**Descending**	**Ectatic**	**Aneurysmal**
	Aortic dilation	3	0	53	21	28	4	2	34	16
	Aortic atherosclerosis	0	0	0	0	0	0	0		
	Aortic arteritis	0	0	0						
Other	Hypertensive pulmonary disease	2	13	6						
	Hypertrophic cardiomyopathy	9	18	85						
	Infiltrative cardiomyopathy	5	5	4						
	Ischemic cardiomyopathy	10	4	22						
	Nonischemic cardiomyopathy	4	12	42						
	Myocardial infarction	3	0	8						
	Myocarditis/pericarditis	32	27	14						
	Papillary muscle thickening	0	0	1						
	Pericardial effusion	5	0	22						
	Pleural effusion	2	0	33						
	Sarcoidosis	11	21	5						
	Amyloidosis	10	13	8						

The number of instances of each of the 34 cardiovascular conditions and their associated features (certainty and severity) are shown. NA denotes attributes that were not applicable to that condition. For some certainty and/or severity levels, 0 indicates that there were no instances of this specific certainty/severity in the entire dataset. *LA* left atrium, *RA* right atrium, *LV* left ventricle, *RV* right ventricle, *CMR* cardiovascular magnetic resonance

We extracted an additional 428 CMR reports from electronic health records from the Cleveland Clinic Florida campus to validate our model on reports from an independent setting from the training set. Half were selected from patients with indications for infiltrative cardiomyopathy and half were randomly selected. We recruited another independent annotator to manually annotate the same cardiovascular conditions and associated attributes on the 428 CMR reports. CMR report similarity between the internal and external validation set was compared using cross-product cosine similarity between the CMR report text.

All CMR reports at the Cleveland Clinic Main Campus were drafted by an advanced imaging fellow first in English and then modified by the staff physician. Half of such staff are radiologists and the other half are cardiologists. There is a high percentage of non-native English speakers, but all of the staff are fluent in English. In contrast, the Cleveland Clinic Florida campus has no advanced imaging fellows. Instead, the MRI reports are dictated directly by staff. The vast majority of such staff are radiologists, with a few cardiologists.

**Fig. 1:**
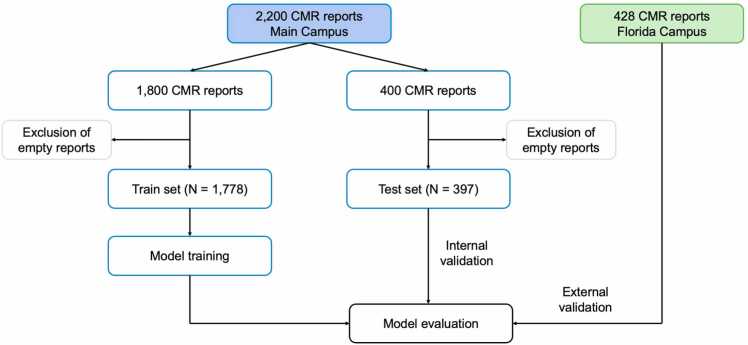
Study workflow. CMR reports were extracted from the electronic health record. A total of 2200 were obtained from the Cleveland Clinic main campus and 428 from the Florida campus. The main campus data was split using a 9:2 ratio and empty CMR reports were excluded. Model was trained and then evaluated on the test set. External validation was performed using the Florida campus data. *CMR* cardiovascular magnetic resonance

In summary, the overall cohort consisted of 1) a training set of 1800 reports which was collectively annotated by one of four annotators, 2) an internal test set of 400 reports which was annotated by all four annotators, and 3) an external test of 428 reports which was annotated by a fifth annotator. Empty reports were excluded from analysis, resulting in 1778 and 397 reports in the training and internal validation set, respectively. No empty reports were found in the external validation set. A schematic of the deep learning network is shown in Fig. 1.

### 2.2 Model training

**Fig. 2:**
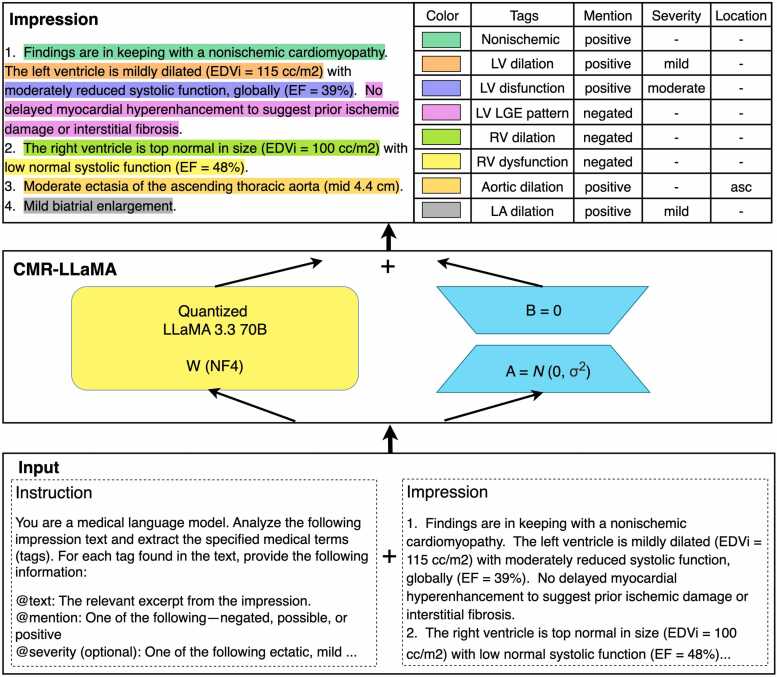
Overview of the CMR-LLaMA pipeline. A structured instruction prompt and impression section of CMR notes are fed into a quantized LLaMA 3.3–70B model fine-tuned via QLoRA. The model then identifies every key finding and extracts its mention status, severity, and anatomical location. *CMR-LLaMA* cardiac magnetic resonance imaging-large language model Meta AI, *QLoRA* quantized low-rank adaptation

We finetuned the LLaMA-3.3–70B-Instruct, a 70-billion parameter, instruction-tuned, auto-regressive language model. We employed 4 bit Quantized Low-Rank Adaptation (QLoRA) to finetune the model. The low-rank adapter was configured with a rank *r* = 8, a scaling factor *α* = 16, and no dropout. Adaptation was applied to all projection layers. Training proceeded with an effective batch size of one via gradient accumulation over 16 steps, starting from an initial learning rate of $3\times 10^{-4}$ which was linearly ramped up over the first 100 steps, and the model was fine-tuned for 1600 steps. For model input, we utilized the impression section of CMR clinical reports, combined with a comprehensive prompt template that provides structured instructions for medical entity extraction. The model then generates JSON-formatted output that captures the cardiovascular conditions. A schematic of our model pipeline is shown in Fig. 2**.**

We also fit two more model classes as a comparison: QwQ-32B from the Qwen series [14] and bidirectional encoder representations from transformers (BERT) [15]. Due to the limited token length that can be accepted by BERT, two versions of this model were created: one with truncation of input text, and another in which CMR reports were split up into sentences and individually inputted into the model.

The language model pre-trained model weights were obtained from HuggingFace [16], [17], [18]. All code was run using Python version 3.9 [19]. The code for this work is located at https://github.com/michelleUMD/cmr-llama.

### 2.3 Evaluation

To evaluate model performance, F1 scores were calculated for each cardiovascular condition as a binary mention (positive/possible vs negated/not mentioned). We chose F1 score over area under the receiver operating characteristic (AUROC) due to AUROC being more subjective to producing deceptively high scores when there are high levels of class imbalance.

In addition to the binary mention of a cardiovascular condition, multiclass F1 scores with micro-averaging were computed for certainty (positive, possible, negated) and severity (mild, mild-moderate, moderate, moderate-severe, severe; as well as ectatic and aneurysmal for aortic dilation).

For left ventricular late gadolinium enhancement pattern (epicardial, mid-myocardial, subendocardial, transmural) and aortic dilation location (root, ascending, descending), as multiple of these attributes being simultaneously true (e.g., dilation of the aortic root and ascending aorta), these attributes were treated as multilabel classification when computing the F1 scores.

## 3 Results

Table 1 contains the number of instances of each condition in the main campus dataset. The most common conditions were left ventricular dilation, left ventricle late gadolinium enhancement, left ventricular dysfunction, aortic dilation, and mitral valve regurgitation. Most valvular pathologies were mild in severity. Aortic dilation was most commonly found in the aortic root and was more likely to be ectatic, rather than aneurysmal. There were select combinations of condition/certainty/severe in which there were 0 instances found in the dataset (e.g., possible and negated right ventricular dilation, moderate-severe aortic valve stenosis).

### 3.1 Inter-annotator agreement

Inter-annotator agreement Fleiss' Kappa Score (κ) scores for each label are displayed in Table 4**.** Scores near or below 0 indicate high levels of disagreement between annotators whereas scores closer to 1.0 indicate high levels of agreement. There was highest agreement in the annotation of the conditions: aortic atherosclerosis (κ = 0.92), tricuspid valve regurgitation (κ = 0.91), and bicuspid aortic valve (κ = 0.88). Left ventricle late gadolinium enhancement and left ventricle fibrosis had the lowest agreement (κ = 0.23 and 0.29, respectively).

**Table 4:** F1 scores for 31 conditions in the internal (main campus) and external (Florida campus) validation sets

	Internal Validation				External Validation			
Condition	Mention	Certainty	Severity	Other	Mention	Certainty	Severity	Other
Mitral Valve Regurgitation	0.97 (0.95–0.99)	0.98 (0.97–0.99)	0.98 (0.96–0.99)		0.95 (0.91–0.98)	0.96 (0.94–0.98)	0.98 (0.96–0.99)	
Aortic Valve Regurgitation	0.99 (0.98–1.00)	0.99 (0.97–1.00)	1.00 (0.99–1.00)		0.94 (0.87–0.99)	0.99 (0.98–1.00)	0.98 (0.96–0.99)	
Aortic Valve Stenosis	0.92 (0.81–1.00)	0.99 (0.98–1.00)	0.99 (0.97–1.00)		1.00 (1.00–1.00)	1.00 (0.99–1.00)	1.00 (0.99–1.00)	
Tricuspid Valve Regurgitation	0.95 (0.89–0.99)	0.99 (0.98–1.00)	0.99 (0.98–1.00)		0.87 (0.75–0.95)	0.98 (0.97–0.99)	0.99 (0.98–1.00)	
Pulmonary Valve Regurgitation	0.92 (0.67–1.00)	1.00 (0.99–1.00)	1.00 (0.99–1.00)		0.92 (0.79–1.00)	1.00 (0.99–1.00)	0.99 (0.97–1.00)	
Aortic Valve Bicuspid	0.91 (0.80–0.98)	0.99 (0.98–1.00)			0.80 (0.44–1.00)	0.99 (0.98–1.00)		
RA Dilation	0.72 (0.58–0.82)	0.94 (0.92–0.96)	0.95 (0.92–0.97)		0.85 (0.71–0.95)	0.98 (0.97–0.99)	0.98 (0.97–1.00)	
LA Dilation	0.94 (0.88–0.98)	0.98 (0.97–0.99)	0.97 (0.96–0.99)		0.88 (0.80–0.96)	0.98 (0.96–0.99)	0.97 (0.96–0.99)	
RV Dysfunction	0.94 (0.89–0.98)	0.98 (0.96–0.99)	0.96 (0.94–0.98)		0.87 (0.81–0.92)	0.95 (0.93–0.97)	0.91 (0.88–0.94)	
RV Dilation	0.98 (0.94–1.00)	0.99 (0.98–1.00)	0.99 (0.97–1.00)		0.96 (0.90–0.99)	0.99 (0.98–1.00)	0.98 (0.96–0.99)	
LV Dysfunction	0.93 (0.90–0.96)	0.96 (0.94–0.98)	0.95 (0.92–0.97)		0.83 (0.75–0.89)	0.94 (0.92–0.96)	0.96 (0.94–0.97)	
LV Dilation	0.93 (0.89–0.95)	0.94 (0.92–0.97)	0.92 (0.90–0.95)		0.89 (0.84–0.92)	0.92 (0.89–0.94)	0.85 (0.82–0.88)	
LV Hypertrophy	0.88 (0.80–0.94)	0.96 (0.95–0.98)	0.97 (0.96–0.99)		0.80 (0.72–0.88)	0.94 (0.91–0.96)	0.91 (0.88–0.94)	
LV Late Gadolinium Enhancement	0.72 (0.65–0.79)	0.85 (0.82–0.89)		0.07 (0.00–0.14)	0.69 (0.61–0.75)	0.81 (0.78–0.85)		0.08 (0.02–0.16)
LV Fibrosis	0.72 (0.57–0.85)	0.96 (0.94–0.98)			0.06 (0.00–0.18)	0.92 (0.90–0.95)		
LV Noncompaction	0.67 (0.00–1.00)	0.99 (0.99–1.00)			0.91 (0.60–1.00)	1.00 (0.99–1.00)		
Aortic Dilation	0.75 (0.40–0.95)	0.99 (0.98–1.00)		0.50 (0.00–0.80)				
Aortic Atherosclerosis	0.97 (0.94–0.99)	0.98 (0.97–0.99)	0.91 (0.88–0.94)	0.92 (0.88–0.95)	0.99 (0.96–1.00)	1.00 (0.99–1.00)	0.97 (0.96–0.99)	0.88 (0.81–0.95)
Aortic Arteritis	0.86 (0.50–1.00)	0.99 (0.99–1.00)						
Hypertensive Pulmonary Disease	0.77 (0.61–0.89)	0.96 (0.95–0.98)			0.85 (0.67–0.96)	0.99 (0.97–1.00)		
Hypertrophic Cardiomyopathy	0.92 (0.84–0.98)	0.99 (0.98–1.00)			0.92 (0.88–0.96)	0.97 (0.95–0.98)		
Infiltrative Cardiomyopathy	0.67 (0.42–0.85)	0.97 (0.95–0.99)			0.50 (0.17–0.74)	0.97 (0.95–0.99)		
Ischemic Cardiomyopathy	0.74 (0.56–0.88)	0.97 (0.95–0.98)			0.76 (0.62–0.87)	0.96 (0.94–0.97)		
Nonischemic Cardiomyopathy	0.82 (0.73–0.89)	0.94 (0.92–0.96)			0.94 (0.89–0.98)	0.98 (0.96–0.99)		
Myocardial Infarction	0.50 (0.29–0.68)	0.94 (0.92–0.96)			0.17 (0.00–0.38)	0.95 (0.93–0.97)		
Myocarditis/Pericarditis	0.92 (0.86–0.96)	0.96 (0.94–0.98)			0.79 (0.67–0.89)	0.96 (0.95–0.98)		
Papillary Muscle Thickening	0.77 (0.52–0.92)	0.98 (0.97–0.99)			0.00 (0.00–0.00)	0.98 (0.97–0.99)		
Pericardial Effusion	0.99 (0.96–1.00)	1.00 (0.99–1.00)			0.90 (0.79–0.98)	0.99 (0.98–1.00)		
Pleural Effusion	0.92 (0.83–0.98)	0.98 (0.97–0.99)			0.95 (0.89–1.00)	0.99 (0.98–1.00)		
Sarcoidosis	0.84 (0.69–0.95)	0.98 (0.96–0.99)			0.86 (0.73–0.96)	0.98 (0.97–0.99)		
Amyloidosis	0.86 (0.62–1.00)	0.99 (0.98–1.00)			0.88 (0.73–0.98)	0.99 (0.97–1.00)		

Micro-average scores are shown for certainty, severity, location, and pattern attributes. 95% confidence intervals are shown in parenthesis. *LA l*eft atrium, *RA* right atrium, *LV* left ventricle, *RV* right ventricle

### 3.2 Model performance

Model weights can be found at https://huggingface.co/michelleUMD/cmr-llama. Hyperparameters used include steps = 1600 steps, beams = 2.

The average F1 for the mention of the 31 conditions was 0.85. The model showed strongest prediction of aortic valve regurgitation (F1 [95% confidence interval (CI)] = 0.99 [ 0.98–1.00]) and right ventricular dilation (F1 [95% CI] = 0.98 [0.94–1.00]) and weakest performance for myocardial infarction (F1 [95% CI] = 0.50 [0.29–0.68]) and left ventricular noncompaction (F1 [95% CI] = 0.67 [0.00–1.00]). There was no significant difference between radiologist (F1 [95% CI] = 0.88 [0.86–0.90]) or cardiologists (F1 [95% CI] = 0.89 [0.87–0.91]) as the reader (Supplemental Figures 2 and 3).

The model showed strong performance for the prediction of condition severity, with F1 ranging between 0.92 [95% CI = 0.90–0.95] for left ventricle dilation to 1.00 [95% CI = 0.90–1.00] for aortic and pulmonary valve regurgitation.

Both the aortic dilation and aortic atherosclerosis conditions had an associated location. The model was able to successfully predict aortic dilation location (F1 [95% CI] = 0.92 [0.88–0.95]) but struggled with atherosclerosis location (F1 [95% CI] = 0.50 [0.00–0.80]). The model also showed weak performance predicting the pattern of left ventricle late gadolinium enhancement (F1 [95% CI] = 0.08 [0.02–0.16]).

**Fig. 3:**
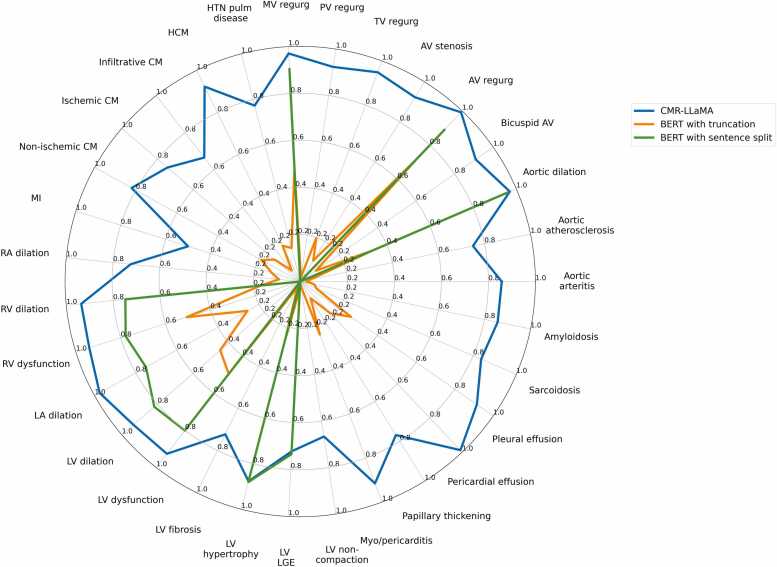
Comparison of CMR-LLaMA, QwQ-32B, and BERT models on the test set. F1 scores are shown for 31 cardiovascular conditions. CMR-LLaMA (shown in blue) was compared against BERT with truncation of input text (red), and BERT with splitting of document input into individual sentences (purple). *CMR-LLaMA* cardiac magnetic resonance imaging-large language model Meta AI, *BERT* bidirectional encoder representations from transformers, *AV* aortic valve, *MV* mitral valve, *PV* pulmonary valve, *TV* tricuspid valve, *LA* left atrium, *RA* right atrium, *LV* left ventricle, *RV* right ventricle, *LGE* late gadolinium enhancement, *CM* cardiomyopathy, *ACS* acute coronary syndrome, *HCM* hypertrophic cardiomyopathy, *MI* myocardial infarction, *HTN* hypertension

Fig. 3 shows a comparison of the F1 scores for the mentions of the 31 cardiovascular conditions between CMR-LLaMA and two BERT models. The QwQ model performed similarly to CMR-LLaMA, so it is not shown. The two BERT models perform significantly worse than CMR-LLaMA.

### 3.3 External validation

Data characteristics of the external validation set can be found in Table 3. There were also generally fewer instances of the 31 cardiovascular conditions in this external validation set compared to the main campus internal validation set. No instances of the aortic atherosclerosis and aortic arteritis conditions were annotated in this new dataset.

On average, the length of the impressions section in the external validation set was shorter (mean = 427; 95% CI 413–441) compared to the main campus test set (mean = 650 95% CI = 618–681). Average string cosine similarity for crosswise string matching was calculated to be 0.73 (95% CI 0.73–0.74). The patient population from which these CMR reports were written about was also markedly different, as shown by the difference in frequencies of the 31 cardiovascular conditions. The ability of our model to have comparable performance on most cardiovascular conditions in this external validation set suggests that our model is generalizable and has broad-scale utility.

Model performance on the external validation set was generally slightly lower than the internal validation set, but performance was still strong. Average F1 score for the mention of the 29 cardiovascular conditions (excluding the aortic atherosclerosis and aortic arteritis conditions with 0 instances) was 0.78. Average certainty F1 was 0.97, and average severity F1 was 0.96. The model performed significantly worse on the left ventricle fibrosis condition (F1 [95% CI] = 0.06 [0.00–0.18] vs 0.72 [0.57–0.85]). The model may also perform slightly better on the external validation set left ventricle noncompaction condition (F1 [95% CI] = 0.91 [0.60–1.00] vs 0.67 [0.00–1.00]).

### 3.4 Failure analysis

A qualitative analysis of the three CMR reports producing the greatest number of model misclassifications for the certainty and severity attributes is shown in Supplemental Table 1**.** Many of these errors stem from human error in the manual annotation process. These manual annotation errors may be attributed to ambiguity in wording such as multi-clause sentences (e.g., “the left ventricle is mildly dilated, and has severely decreased function”) or non-specific wording (e.g., “there is no significant atrial enlargement”).

## 4 Discussion

This study presents a model trained to extract 31 cardiovascular conditions and their associated attributes from CMR reports. Our model shows strong performance in identifying select cardiac valve, chamber, and aortic pathologies. This enables efficient, automated identification of a number of medical diagnoses and characterization of a patient’s morphology, function, and myocardial viability. The model is additionally able to provide qualitative attributes about these conditions, including certainty, severity, and location.

Applying this model to clinical practice can help lay the foundation for future clinical decision support tools. Many risk models (e.g., sudden cardiac death risk calculator) include information contained within free-text notes, such as the extent of late gadolinium enhancement (LGE). This model offers the potential to quickly parse CMR reports into common data elements [20]. This discretization of data enables the automation of such models, reducing provider burden and streamlining workflow [21]. Furthermore, it enables the data to be searchable in common structured query language (SQL) frameworks, thereby augmenting the value of CMR studies for both decision making, operations, and research. Applying this model broadly on a cohort level can expedite the collection of research databases across different studies and sites in a consistent and reproducible manner. This is essential for multicenter trials and registries, where imaging data is often used for both endpoints and patient characterization. Generation of such databases opens up numerous possibilities for downstream analysis, including ground-truth labeling of CMR images for computer vision studies.

To the best of our knowledge, this is the largest study to have developed such a language model for CMR reports, with a total of 2175 reports being used. The model we present here also is able to recognize the most number of cardiovascular conditions, trained to extract 31 cardiovascular conditions, while other studies have only looked at only one [9] or five diagnoses [10]. Previous studies have treated cardiovascular conditions as binary conditions (present and not present) [10], whereas the present study is able to capture multiclass certainty (negated, possible, positive) and severity (mild, mild-moderate, moderate, moderate-severe, severe), along with other attributes relating to the severity, subtype, and location of the cardiovascular condition. Another advantage of CMR-LLaMA is its ability to input relatively long input text, allowing for input of the entire CMR impressions section without need for loss of data through truncation, which a prior study faced using a BERT model [10].

The model tended to perform the best on conditions with high number of occurrences and high inter-annotator agreement in the test set. For example, mitral and aortic valve regurgitation had some of the highest number of occurrences, κ agreement scores, and F1 model performances amongst all the other cardiovascular conditions. The model showed poor performance in predicting late gadolinium enhancement pattern. The ability of CMR to visualize late gadolinium enhancement is one of the main uses of this imaging modality. Left ventricle late gadolinium enhancement was mentioned 688 times in the main campus dataset, but κ agreement score was only 0.23 for its mention and 0.25 for the actual pattern of enhancement. This low level of agreement likely contributed to the model’s poor performance of this condition. The high variability between human annotators suggests that documentation of this condition is highly subjective and further emphasizes the need for such a model to standardize data extraction. Our study has several limitations. One limitation of our current model is a lack of standardized language for describing imaging findings. Standard ontologies used in imaging such as Systematized Nomenclature of Medicine-Clinical Terms (SNOMED-CT) [22] promote clarity and improve data exchange between systems. Modifying our model to extract data in a common lexicon could be an ideal next step for improving interoperability of our model’s output and enabling integration with structured reporting systems such as Digital Imaging and Communications in Medicine (DICOM) and Health Level 7 (HL7). Second, although we validated the model at two vastly different sites, the model was also only validated within a single healthcare system. Further validation at other healthcare systems is warranted. Finally, the annotation process was done by medical students and one resident physician, and discrepancies between annotators were reconciled using a voting system, rather than having an individual with higher level training manually reconcile discrepancies due to limitation in human resources. In summary, we developed a large language models to identify cardiovascular conditions and their certainty, severity, location, and subtype from patient CMR reports. By applying these models, we can automate and increase the efficiency of this data extraction process. This extracted data can then be used to correlate with and complement other data extracted from imaging and billing codes to improve healthcare outcomes and enhance the quality of care.

## Author contributions

**Michelle Z. Fang: Conceptualization, Data curation,** Formal analysis, Investigation, Validation, Visualization, Writing – original draft. **Makiya Nakashima:** Data curation, Formal analysis, Investigation, Methodology, Software, Validation, Writing – review & editing. **Kailash Singh:** Data curation, Formal analysis, Validation, Writing – review & editing. **Eileen Galvani:** Data curation, Investigation. **Xiaotan Sun:** Formal analysis, Investigation, Methodology, Validation. **Sharmeen Sorathia:** Data curation, Formal analysis, Investigation. **Kevin Dorocak:** Data curation, Formal analysis. **Deborah Kwon:** Conceptualization, Funding acquisition, Resources, Supervision, Writing – review & editing. **Christopher Nguyen:** Conceptualization, Resources, Supervision, Writing – review & editing. **David Chen:** Conceptualization, Formal analysis, Investigation, Methodology, Project administration, Resources, Supervision, Writing – review & editing.

## Declaration of competing interests

The authors declare the following financial interests/personal relationships which may be considered as potential competing interests. Deborah Kwon reports a relationship with Society for Cardiovascular Magnetic Resonance that includes board membership. Other authors declare that they have no known competing financial interests or personal relationships that could have appeared to influence the work reported in this paper.

## References

[1] Marcu C.B., Beek A.M., van Rossum A.C. (2006). Clinical applications of cardiovascular magnetic resonance imaging. CMAJ Can Med Assoc J.

[2] Karamitsos T.D., Francis J.M., Myerson S., Selvanayagam J.B., Neubauer S. (2009). The role of cardiovascular magnetic resonance imaging in heart failure. J Am Coll Cardiol.

[3] Myerson S.G. (2021). CMR in evaluating valvular heart disease. JACC Cardiovasc Imaging.

[4] Emrich T., Halfmann M., Schoepf U.J., Kreitner K.-F. (2021). CMR for myocardial characterization in ischemic heart disease: state-of-the-art and future developments. Eur Radiol Exp.

[5] Ebbers T., Kool R.B., Smeele L.E., Dirven R., Den Besten C.A., Karssemakers L.H.E. (2022). The impact of structured and standardized documentation on documentation quality; a multicenter, retrospective study. J Med Syst.

[6] Rizkallah D., Greenberg N.L., Khurana R., Palanisamy V., Alencherry B., Ammoury C. (2025). Impact of automated transfer of semi-automated segmentation and structured report rule requirements on cardiac MRI report quality, standardization, and efficiency. AMIA Annu Symp Proc.

[7] Monda E., Limongelli G. (2023). Integrated sudden cardiac death risk prediction model for patients with hypertrophic cardiomyopathy. Circulation.

[8] Shojania K.G., Jennings A., Mayhew A., Ramsay C., Eccles M., Grimshaw J. (2010). Effect of point-of-care computer reminders on physician behaviour: a systematic review. CMAJ.

[9] Dewaswala N., Chen D., Bhopalwala H., Kaggal V.C., Murphy S.P., Bos J.M. (2022). Natural language processing for identification of hypertrophic cardiomyopathy patients from cardiac magnetic resonance reports. BMC Med Inf Decis Mak.

[10] Zaman S., Petri C., Vimalesvaran K., Howard J., Bharath A., Francis D. (2022). Automatic diagnosis labeling of cardiovascular MRI by using semisupervised natural language processing of text reports. Radiol Artif Intell.

[11] Sundaram D.S.B., Arunachalam S.P., Damani D.N., Farahani N.Z., Enayati M., Pasupathy K.S. (2021). Natural language processing based machine learning model using cardiac MRI reports to identify hypertrophic cardiomyopathy patients. Proc Des Med Devices Conf.

[12] Radiology (ACR) RS of NA (RSNA) and AC of. All About Your Radiology Report: What to Know [Internet]. Radiologyinfo.org. [cited 2025 Jul 2]. Available from: 〈https://www.radiologyinfo.org/en/info/all-about-your-radiology-report〉.

[13] He H., Fu S., Wang L., Liu S., Wen A., Liu H. (2022). MedTator: a serverless annotation tool for corpus development. Bioinformatics.

[14] Peng B., Quesnelle J., Fan H., Shippole E. YaRN: Efficient Context Window Extension of Large Language Models [Internet]. arXiv; 2023 [cited 2025 Jun 22]. Available from: 〈http://arxiv.org/abs/2309.00071〉.

[15] Devlin J., Chang M.-W., Lee K., Toutanova K. BERT: Pre-training of Deep Bidirectional Transformers for Language Understanding [Internet]. arXiv; 2019 [cited 2025 Jun 22]. Available from: 〈http://arxiv.org/abs/1810.04805〉.

[16] meta-llama (Meta Llama) [Internet]. 2025 [cited 2025 Jun 22]. Available from: 〈https://huggingface.co/meta-llama〉.

[17] Qwen/QwQ-32B·Hugging Face [Internet]. 2025 [cited 2025 Jun 22]. Available from: 〈https://huggingface.co/Qwen/QwQ-32B〉.

[18] Paper page - BERT: Pre-training of Deep Bidirectional Transformers for Language Understanding [Internet]. 2024 [cited 2025 Jun 22]. Available from: 〈https://huggingface.co/papers/1810.04805〉.

[19] Ansel J., Yang E., He H., Gimelshein N., Jain A., Voznesensky M., et al. PyTorch 2: Faster Machine Learning Through Dynamic Python Bytecode Transformation and Graph Compilation. Proc 29th ACM Int Conf Archit Support Program Lang Oper Syst Vol 2 [Internet]. La Jolla CA USA: ACM; 2024 [cited 2024 Jun 17]. p. 929–947. Available from: 〈https://dl.acm.org/doi/10.1145/3620665.3640366〉.

[20] Sheehan J., Hirschfeld S., Foster E., Ghitza U., Goetz K., Karpinski J. (2016). Improving the value of clinical research through the use of common data elements. Clin Trials.

[21] Ahmadian L., van Engen-Verheul M., Bakhshi-Raiez F., Peek N., Cornet R., de Keizer N.F. (2011). The role of standardized data and terminological systems in computerized clinical decision support systems: literature review and survey. Int J Med Inf.

[22] Donnelly K. (2006). SNOMED-CT: the advanced terminology and coding system for eHealth. Stud Health Technol Inf.

