# Supplementary material for: Cardiac magnetic resonance imaging-large language model Meta AI: a finetuned large language model for identifying findings and associated attributes in cardiac magnetic resonance imaging reports
Source: J Cardiovasc Magn Reson. 2025 Nov 13;27(2):101968. doi: 10.1016/j.jocmr.2025.101968 (PMC12766592; doi:10.1016/j.jocmr.2025.101968)
Supplement: Supplementary file 1 — Supplementary material [file mmc1.docx]

# Supplemental

**Supplemental Figure 1.** A hypothetical example of an annotated cardiac MRI report.

**
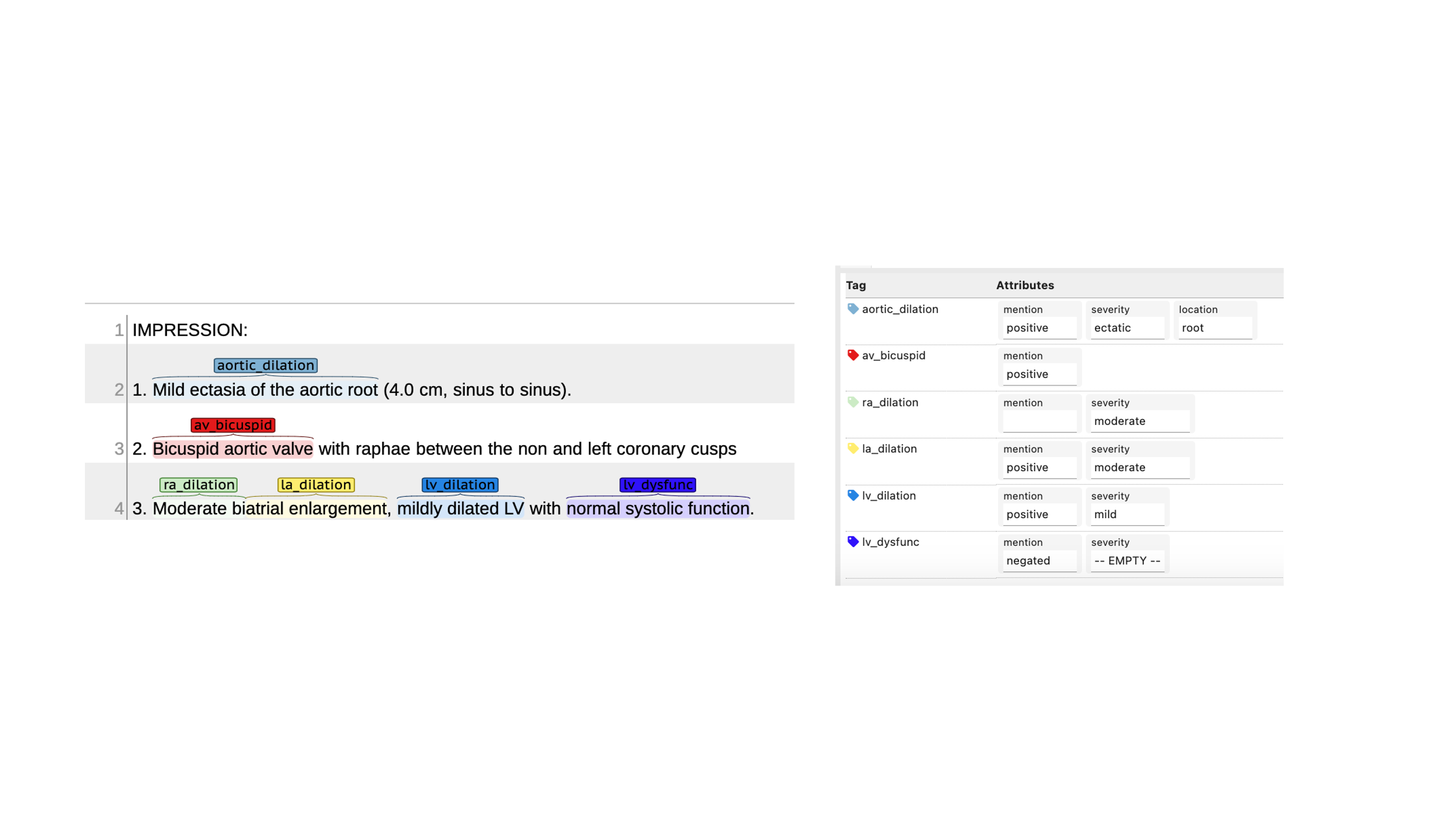
**

**Supplemental Figure 2.** Comparison of CMR-LLaMA performance between radiologists and cardiologist on studies read at Main Campus.


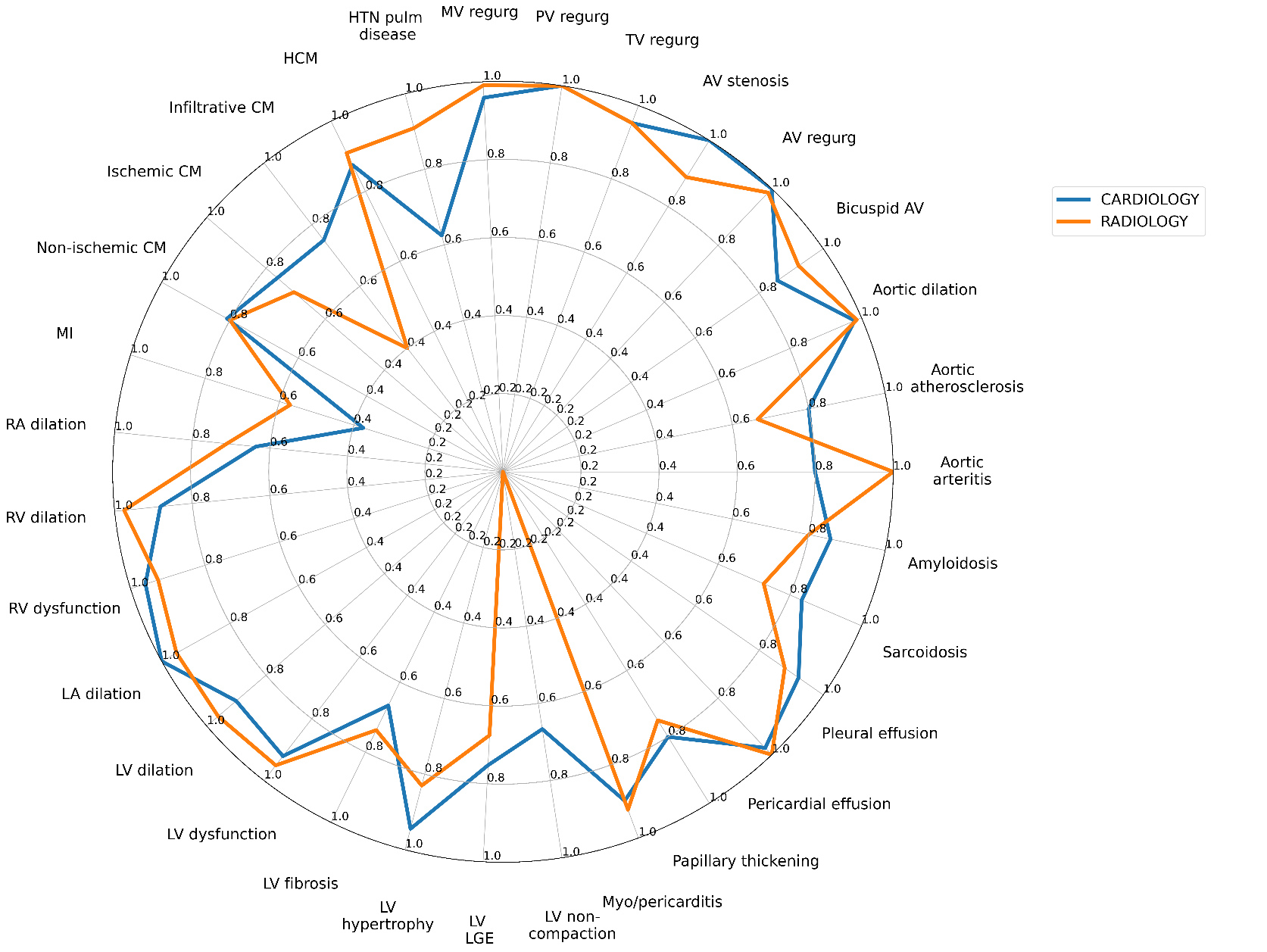


**Supplemental Figure 3.** Comparison of CMR-LLaMA performance between radiologists and cardiologist on studies read at Florida campus.


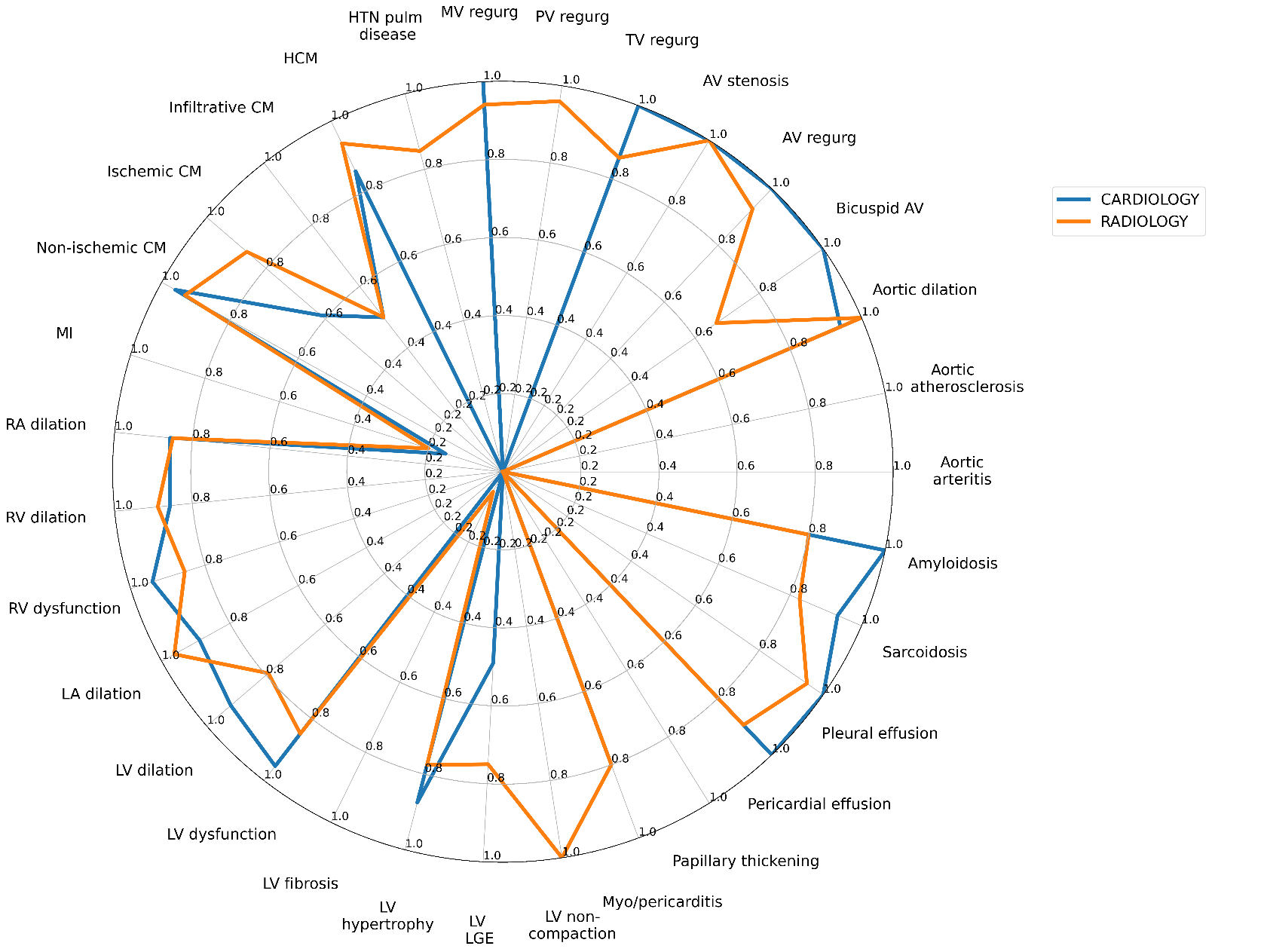


**Supplemental Table 1. Examples of model failures.** The top 3 CMR reports producing the most model error were selected and the incorrect predictions are shown below. Reports were then reviewed by an independent trained reviewer. Red text indicates conditions the independent reviewer indicated that the model prediction was correct, whereas the voted value between the 4 human annotators was not.

| **Text** | **Condition** | **True** | **Predicted** |
| --- | --- | --- | --- |
| IMPRESSION: 1. The left ventricle is severely dilated with severe systolic dysfunction. 2. The calculated left ventricular ejection fraction is 24%. There is global hypokinesis with superimposed regional abnormalities that are worse in the mid, distal anterior, anteroapical, and anterolateral walls of the left ventricle. A large nonmobile apical thrombus measuring 2.7 x 1.5 cm is seen on late gadolinium enhancement sequences. 3. T1 mapping of the myocardium indicated a global native T1 value of 998+/-82 ms (normal values for this scanner 945 - 965 ms, SHMOLLI). Late gadolinium enhancement sequences indicated the presence of a near transmural scar (representing a prior myocardial infarction) involving the distal anterior, apical walls of the left ventricle. The apex is nonviable. There is also a medium-sized scar involving the anterolateral wall of the left ventricle. The distal anterolateral wall demonstrates partial viability. The scar occupies more than 50% of the myocardial thickness in the basal, mid anterolateral walls; and these walls are considered nonviable. The inferior wall is fully viable. The basal, mid anterior walls are also viable. 4. The right ventricle is moderately dilated with severe systolic dysfunction.,The tricuspid annular excursion is measured at 0.9 cm. The right ventricle ejection fraction is calculated at 24% 5. Severe tethering of the mitral valve leaflets is evident, along with annular dilatation. There is moderate central mitral regurgitation. Using phase velocity mapping sequences, the regurgitant volume is calculated at 23 cc, the regurgitant fraction is calculated at 30%. 6. The pericardial thickness is 1 mm. A small pericardial effusion is present without changes of tamponade. 7. The great vessels including the aorta, main pulmonary artery, superior and inferior vena cavae and pulmonary veins are within normal limits. The cardiac situs is solitus. The atrioventricular, ventricular arterial connections appear to be normal. Mild mediastinal lymphadenopathy is evident. No significant intracardiac shunting is observed. The QP/QS ratio is calculated at 0.97  MEASUREMENTS: A. GREAT VESSELS: I. Aortic root 3.5 cm at the sinuses of Valsalva, 2.9 cm at the sinotubular junction. II. Ascending Aorta 3.4 cm | LV dilation | 0 | 1 |
|  | LV dysfunction | 0 | 1 |
|  | LV late gadolinium enhancement | 0 | 1 |
|  | Mitral valve regurgitation | 0 | 1 |
|  | Non-ischemic cardiomyopathy | 0 | 1 |
|  | Sarcoidosis | 0 | 1 |
| IMPRESSION: 1. The left ventricle is normal in size, with low normal systolic function. 2. The calculated left ventricular ejection fraction is 54%. There are no regional wall motion abnormalities. 3. T1 mapping of the myocardium indicated a global native T1 value of 953+/-21 ms (normal values for this scanner 945 - 965 ms, SHMOLLI). Late gadolinium enhancement sequences indicated no evidence of scars involving the left or right ventricles. T2-weighted STIR sequences indicated mild myocardial edema. The signal intensity ratio of the myocardium to skeletal muscle was calculated at 2.9. The T2 value of the native myocardium is calculated at 49 +/-8 ms (normal values for this scanner 40 to 45 milliseconds). By itself, this finding is nonspecific, and should not be used in isolation for diagnosis of myocarditis. Only one out of 3 modified Lake Louise criteria are seen on the study, and this decreases the specificity of the diagnosis of myocarditis. 4. The right ventricle is normal in size, with normal systolic function. The tricuspid annular excursion is measured at 1.7 cm. The right ventricular ejection fraction is calculated at 48% 5. No significant valvular abnormalities seen.. There is no significant atrial enlargement. 6. The pericardial thickness is 1 mm. A trivial (physiologic) pericardial effusion is present without changes of tamponade. 7. The great vessels including the aorta, main pulmonary artery, superior and inferior vena cavae and pulmonary veins are within normal limits. The cardiac situs is solitus. The venoatrial, atrioventricular, ventricular arterial connections appear to be concordant. 8. No significant mediastinal lymphadenopathy is seen. Bilateral breast implants are seen. 9. Using phase velocity mapping sequences, there is no evidence of significant intracardiac shunting. The QP/QS ratio is calculated at 0.99  MEASUREMENTS: A. GREAT VESSELS: I. Aortic root 3.1 cm at the sinuses of Valsalva, 2.3 cm at the sinotubular junction II. Ascending Aorta 2.5 cm | Amyloidosis | 1 | 0 |
|  | Aortic valve regurgitation | 1 | 0 |
|  | Hypertrophic cardiomyopathy | 1 | 0 |
|  | Non-ischemic cardiomyopathy | 1 | 1 |
|  | RA dilation | 1 | 0 |
|  | Sarcoidosis | 1 | 0 |
| IMPRESSION: * Moderate biatrial enlargement. * There is flow across the interatrial septum, predominantly left to right. The defect is not clearly demarcated but is likely subcentimeter in size based upon adjacent flow jets. Findings in keeping with known ASD (Calculated Qp/Qs: 2.7). * The left ventricle is mildly dilated, and has severely decreased function (EDVi = 105 cc/m2, LVEF = 18 %). There are nonspecific patchy mid-myocardial delayed enhancement in the mid inferior RV insertion extending to the septum in a non-ischemic pattern, this is nonspecific and can be seen in setting of ventricular pressure overload. * The right ventricle is normal in size and shape, and has moderately decreased function (RVEF = 32 %). * There is trivial visualized aortic regurgitation (Aortic regurgitant fraction: 5%) * There is trivial visualized pulmonic insufficiency (Pulmonic regurgitant fraction: 6%) * There is mild mitral regurgitation by visual assessment (Quantitative mitral regurgitant fraction: 23%) * The thoracic aorta appears normal in course, caliber, and contour. * Trace bilateral pleural effusion. There is a small pericardial effusion. * The pulmonary arteries are dilated (main PA: 3.6 cm). | Amyloidosis | 1 | 0 |
|  | Aortic dilation | 1 | 1 |
|  | Infiltrative cardiomyopathy | 1 | 1 |
|  | LV dysfunction | 0 | 1 |
|  | LV fibrosis | 1 | 0 |
|  | LV late gadolinium enhancement | 1 | 0 |
