# Supplementary figures and images for: Cardiac magnetic resonance imaging-large language model Meta AI: a finetuned large language model for identifying findings and associated attributes in cardiac magnetic resonance imaging reports
Source: J Cardiovasc Magn Reson. 2025 Nov 13;27(2):101968. doi: 10.1016/j.jocmr.2025.101968 (PMC12766592; doi:10.1016/j.jocmr.2025.101968)

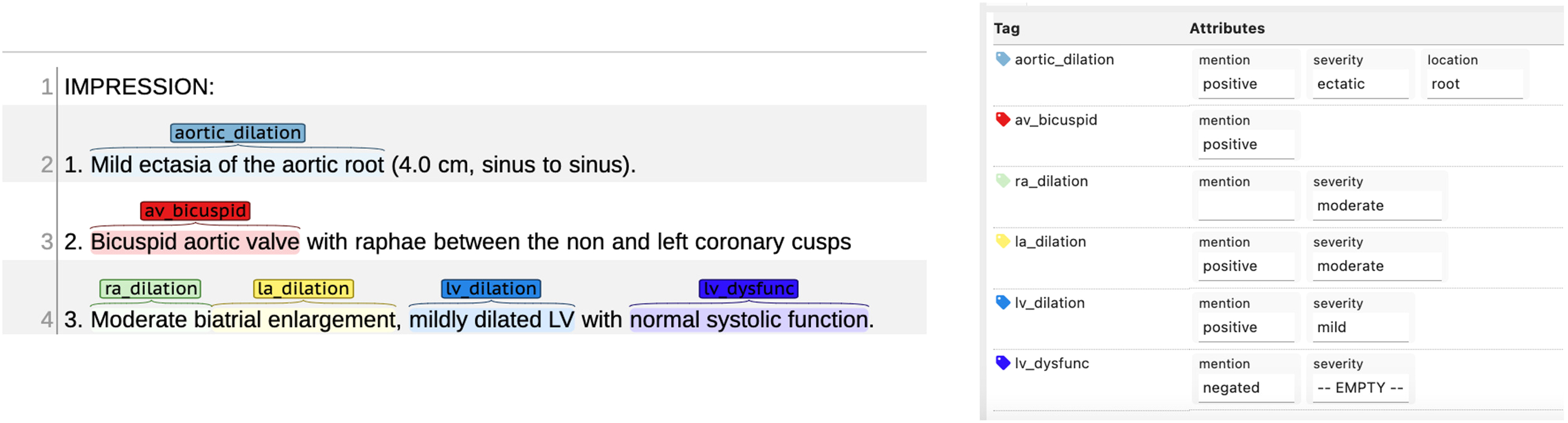

Supplement: Supplementary file 2 — Supplementary material [file mmc2.jpg]
